# Long-Term Side Effects after Treatment in Young Adult Survivors of Childhood Cancer

**DOI:** 10.1080/135771402753667709

**Published:** 2002-05


### LONG-TERM SIDE EFFECTS AFTER TREATMENT IN YOUNG ADULT SURVIVORS OF CHILDHOOD CANCER

### ORAL PRESENTATIONS

### G127 Quality of Life in Young Adults Who are Long-term Survivors of Childhood Bone Cancer

N.E. Langeveld
*Late Effect Study Group, Department of Paediatric Oncology, Emma Kinderziekenhuis AMC, Academic Medical Center, Meibergdreef 9, 1105 AZ, Amsterdam, The Netherlands*

*Objectives:* Although the treatment results of childhood cancer have improved over the last 20 years, emerging reports on late effects of both the disease and the therapy have raised serious concerns about the quality of life of the survivors. While sophisticated data on specific problems are available, very little is known about the overall quality of life of the patients surviving bone cancer into adulthood. As part of a large follow-up project among adult survivors of childhood cancer, a study was done to assess the quality of life in a group of long-term survivors of childhood bone cancer. The results were compared with the quality of life of an age-matched comparison group of persons with no history of cancer.
*Methods:* Participants were 49 survivors of bone cancer (Ewing's sarcoma n=20, osteosarcoma n=29; age range 18–39 years; 51% female) who completed treatment an average of 13 years previously and 1092 persons (age range 15–53 years, 54% female) with no history of cancer. All participants completed a self-report questionnaire probing aspects of quality of life.
*Results:* Survivors and controls did not differ in educational achievement and employment status, but survivors were less likely to be married or living together ($p < 0.05$). There were no statistical differences between the two groups in terms of depression, self-esteem and fatigue. In comparison with the controls, survivors reported significantly more restrictions in physical functioning (primarily in strenuous levels of activity, like running, participating in strenuous sports and climbing stairs), but significantly higher levels of vitality and better general health perceptions.
*Conclusions:* In general, the quality of life in young adult survivors of childhood bone cancer was quite satisfactory. However, some problems were identified and additional research is necessary to define those populations at greatest risk.

### G128 The Hidden Long-term Effects of Treatment

V. Riley
*Bone and Soft Tissue Sarcoma Service, The Middlesex Hospital, London, United Kingdom*

As more young people with cancer are successfully treated, the long term psychological effects are becoming increasingly apparent. Whilst the physical side effects of treatment cannot be overlooked, there are significant long-term psychological issues that are equally important. At the end of treatment most adolescents and young people strive to return to their previous life in the desire to put their cancer experience behind them. The difficulties of achieving this cannot be underestimated as the majority will recognise that they have been through a life changing experience. However, many do manage to achieve their goals but at some point many cancer survivors will begin a painful re-assessment of who they are. At the completion of treatment the most important issue is the desire to be cured but as time passes the cost cure emerges as a significant issue. A 14 year old being told they are infertile when they are fighting for their life, is very different to a young person in a long-term relationship who has been off treatment for a number of years. A 16 year old who has a hind-quarter amputation for extensive disease has very different issues to 23 year old amputee cancer-survivor. Survivorship guilt issues will effect perceptions of relationships and life achievements. As the long term psychological effects of cancer and its treatment become apparent it is vital that health care professionals recognise these issues. Whilst the physical well being of patients is monitored through long-term follow up, it is equally important that the long term psychological effects are identified. Research is vital if these issues are to gain recognition and respect, and then the appropriate support can be made available.

### G129 Functional Outcome Following Endo-Prosthetic Replacement (EPR) for Bone Tumours Involving the Knee

C. Williams, J. Davies
*Royal Orthopaedic Hospital NHS Trust, Birmingham, United Kingdom*

*Background:* Since the introduction of adjuvant therapies to the treatment of bone tumours, rehabilitation is becoming more of a focus due to an increase in survival rate. In order to investigate the outcome of surgery and rehabilitation needs of patients undergoing endo-prosthetic replacement (EPR), functional analysis and long-term follow-up is being undertaken. This abstract presents short-term results only as the study is ongoing.
*Aims:* To ascertain the level of function of patients undergoing distal femoral and proximal tibial EPRs for the treatment of bone tumours and how this changes following surgery and rehabilitation.
*Method:* Patients are initially seen pre-operatively in order to gain a baseline score, then followed up for 18 months. Patients completed 2 questionnaires (1) Toronto Extremity Salvage Score (TESS) and (2) Euroquol Health Score. Data is entered into an access database.
*Results:* To date 27 patients have been recruited. Average age is 43 (20–71), 18 men and 9 women.
TESS scores
Distal femoral EPR (15) Pre-op 53%, 6 weeks 58%, 4.5 months 69%
Proximal Femoral EPR (12) Pre-op 41%, 6 weeks 46%, 4.5 months 63%
*Conclusion:* Early results indicate that physical function improves quickly following surgery and that this improvement is maintained. Clinical implications of these early findings will be discussed and data presented regarding quality of life.

